# Biatrial myxoma floating like a butterfly: A case report and review of the literature

**DOI:** 10.1097/MD.0000000000009558

**Published:** 2018-01-19

**Authors:** Yanhui Li, Xiaodong Li, Xiaocong Wang, Liping Chen

**Affiliations:** Department of Echocardiography, Center of Cardiovascular diseases, The First Hospital of Jilin University, Changchun, China.

**Keywords:** biatrial, echocardiography, myxoma

## Abstract

**Rationale:**

Myxoma is the most common type of primary benign cardiac tumor in adults. The left atrium is the most frequent site of origin followed by the right atrium. Biatrial myxoma is extremely rare.

**Patient concerns:**

We present a case of a 60-year-old woman with biatrial myxoma, who presented with palpitations for one month.

**Diagnoses:**

Echocardiography revealed an irregular homogeneous mass in the left atrium and in the right atrium, and were connected via the fossa ovalis suspiciously. Computed tomography angiography revealed a hypo-intense mass in both atria.

**Interventions:**

The tumors were successfully removed by surgical excision and histological analysis confirmed the diagnosis.

**Outcomes:**

The patient was discharged one week after surgery, and did not experience recurrence during the two years follow-up period.

**Lessons:**

Biatrial myxoma is rare. Surgical resection is the mainstay of treatment and there is no recurrence reported. The clinical data and the features on echocardiogram of biatrial myxoma are reviewed, providing important clinical information for the pre-operative diagnosis and intraoperative removal of biatrial myxoma.

## 1 Introduction

Myxoma is the most common primary cardiac tumor with an estimated incidence of between 8 and 150 cases per million.^[1]^ The left atrium is the most common site of origin (60%–88%), and in very rare cases, myxomas are found in both atria in the same patient (<2.5%).^[2]^ Here, we present a patient with a biatrial myxoma as an incidental finding on echocardiography who underwent successful surgery.

## 2 Case presentation

A 60-year-old woman was admitted to our hospital complaining of palpitations for 1 month. There was no history of cardiac disease. On physical examination, the heart rate was regular at 85 beats/min and blood pressure was 107/65 mm Hg. Auscultation revealed no systolic or diastolic murmur. Electrocardiogram showed a normal sinus rhythm. Chest x-ray revealed no pulmonary disease. Laboratory analyses were normal.

**Figure 1:**
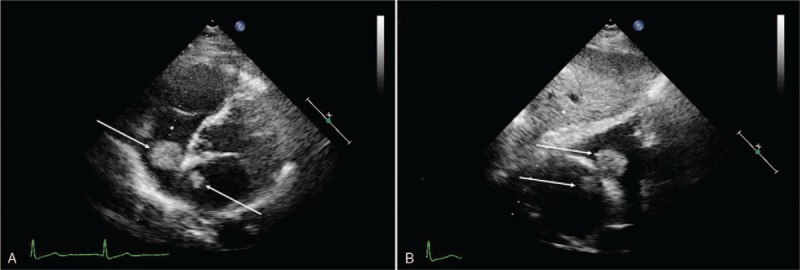
Parasternal 4-chamber view (A) and subcostal view (B) on transthoracic echocardiogram visualization of the biatrial tumor. The white arrow showing the myxoma in the right atrium and the left atrium.

**Figure 2:**
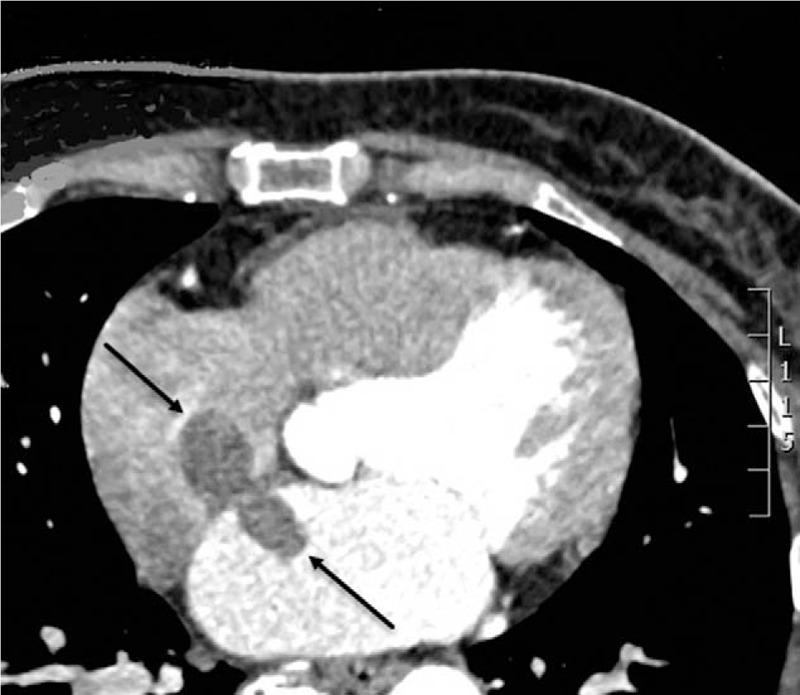
A. computed tomography scan showing a mass in both atria. The black arrow shows the myxoma in the right atrium and the left atrium.

Transthoracic echocardiography revealed an irregular homogeneous mass in the left atrium measured approximately 23 × 11 mm originating from the left side of the interatrial septum (IAS) and attached to the IAS by a stalk. Another similar echogenic mass in the right atrium which measured approximately 23 × 19 mm was attached to the right side of IAS (Fig. 1A and B). Both the left and right masses were located opposite to each other, floating simultaneously like a butterfly during the cycle and did not protrude into the atrioventricular valve during diastole. Based on the above findings, we diagnosed that biatrial myxoma and the tumors were connected via the fossa ovalis. Computed tomography angiography (CTA) revealed no significant coronary artery stenosis, but a hypointense mass in both atria (Fig. 2).

**Figure 3:**
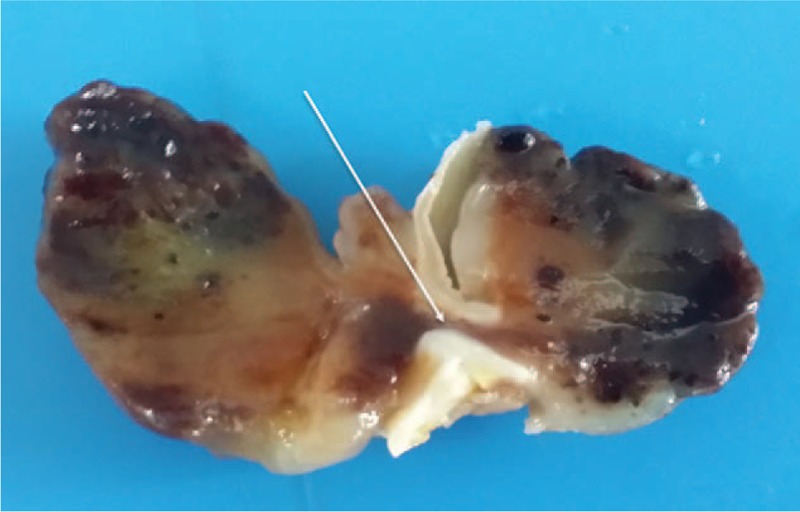
Gross appearance showing that the tumor was connected via the fossa ovalis. The white arrow shows the fossa ovalis.

**Figure 4:**
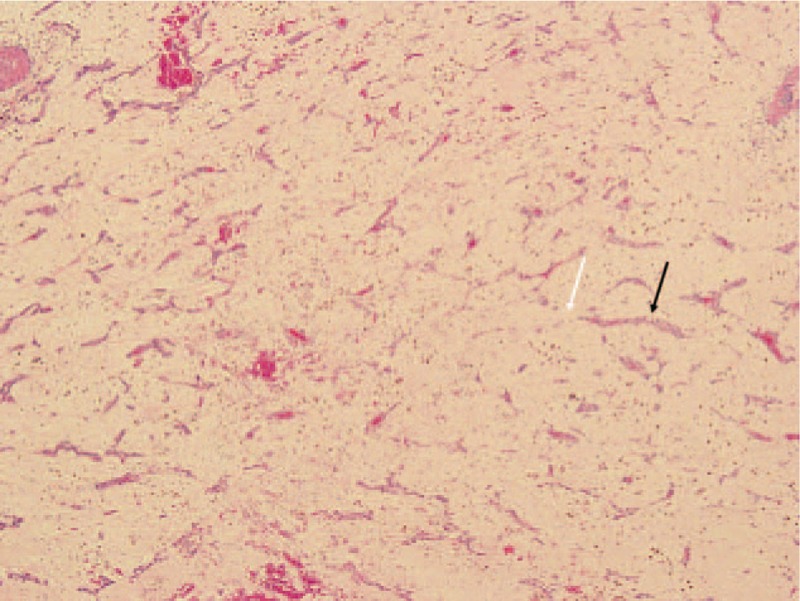
Biatrial tumor which was myxoma by pathological examination (H&E ×40). Photomicrograph of the excised mass showing cords of tumor cells (black arrow) with eosinophilic cytoplasm. The tumor cells were within an abundant myxoid stroma (white arrow).

Surgery was performed under a standard cardiopulmonary bypass. The tumors were found to be soft and fragile. The left and right atrial myxomas were completely removed and the attached atrial septum was resected; the interatrial residual defect was closed with a 4-0 Prolene running suture. Pathology showed that the excised masses were connected via the fossa ovalis (Fig. 3). Histological examination showed features typical of tumor cells containing eosinophilic cytoplasm within an abundant myxoid stroma (Fig. 4) and confirmed the diagnosis of myxoma. The patient was discharged 1 week after surgery, and did not experience recurrence during the 2-year follow-up period.

## 3 Discussion

Most myxomas are found in the left atrium (60%–88%), with a small percentage in the right atrium (4%–28%), left ventricle (8%), right ventricle (2.5%–6.1%), and in both atria of the same patient (<2.5%).^[2]^ A review by Irani et al^[3]^ in 2008 showed that 11 cases of biatrial myxoma had been reported in the literature since 1998 (including Irani's report). Our case is the 21st biatrial myxoma reported since 2008, and our review of the English literature revealed 20 other entries since 2008 (including Irani‘s report) and describes a total of 20 patients and the details of these biatrial myxomas (Table 1).

**Table 1:**
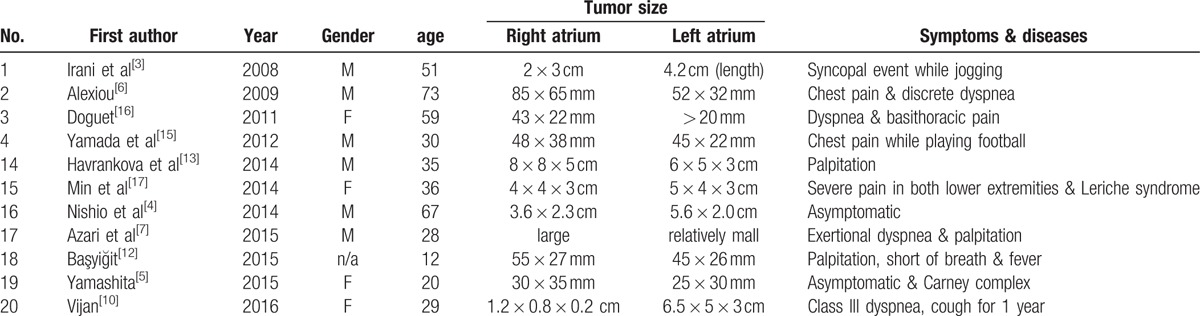
Clinical summary of biatrial myxomas cases found in English literature since 2008.

The symptoms of reported biatrial myxomas vary. In this review, 2 patients were asymptomatic;^[4,5]^ however, symptoms were present in the other patients. The most common symptom in our review was dyspnea or exertional dyspnea,^[6–10]^ followed by syncope^[3,11]^ and palpitation.^[12,13]^ Two patients complained of cough^[10,14]^ and 1 complained of chest pain.^[15]^

Most patients had symptoms of cardiac disease. Therefore, echocardiography is necessary and serial views are needed to evaluate the tumor. A subcostal view is strongly recommended as the interatrial septum is clearly shown and the masses in the 2 atria are visible.

As the extensions of the myxoma are gelatinous and fragile, they tend to break into pieces;^[2]^ cardiac myxomas may have a role in systemic and pulmonary embolisms. In our review, 1 patient was diagnosed with myxoma and pulmonary embolism;^[16]^ another patient had myxoma combined with infarction of multiple organs.^[17]^ Therefore, once the diagnosis is confirmed, surgical excision of cardiac myxoma is required as soon as possible. Although our patient did not have obvious symptoms, and the tumors were small in size and did not obstruct the mitral and tricuspid valves, surgical removal of the masses was necessary and was quickly performed. No recurrence of myxoma was observed during the 2-year follow-up period.

## 4 Conclusion

Biatrial myxoma is relatively rare and a connection via the fossa ovalis is extremely rare. To our knowledge, our patient is the 21st case of biatrial myxoma reported since 2008. The diagnosis may be established with multiple modalities; however, echocardiography is the initial imaging modality of choice to evaluate the cardiac mass. Although the recurrence rate is relatively low, patients should be followed with serial echocardiography monitoring.

## Acknowledgments

The authors would like to acknowledge the patient and her family for allowing them to use her medical records in their case report and allowing this case to be published.
